# Kdm6a suppresses the alternative activation of macrophages and impairs energy expenditure in obesity

**DOI:** 10.1038/s41418-020-00694-8

**Published:** 2020-12-10

**Authors:** Jun Chen, Xing Xu, Yan Li, Fan Li, Jianjun Zhang, Qin Xu, Wantao Chen, Yan Wei, Xu Wang

**Affiliations:** 1 Department of Oral and Maxillofacial-Head and Neck Oncology, Shanghai Ninth People’s Hospital, Shanghai Jiao Tong University School of Medicine, Shanghai, 200011 China; 2 National Clinical Research Center for Oral Disease, Shanghai, 200011 China; 3 Shanghai Key Laboratory of Stomatology & Shanghai Research Institute of Stomatology, Shanghai, 200011 China; 4 Eye Institute and Department of Ophthalmology, Eye & ENT Hospital, Fudan University, Shanghai, 200031 China; 5 Department of Ophthalmology, Shanghai Xinhua Hospital, Shanghai Jiao Tong University School of Medicine, Shanghai, 200092 China

**Keywords:** Interleukins, Epigenetics

## Abstract

Histone lysine demethylase 6a (Kdm6a) mediates the removal of repressive trimethylation from histone H3 lysine 27 (H3K27me3) to activate target gene expression. Obesity is associated with metabolic inflammation, and adipose tissue macrophages (ATMs) are key players orchestrating metabolic inflammation. However, it is still unclear whether the Kdm6a pathway in ATMs regulates energy homeostasis. Here, we identified Kdm6a as a critical epigenetic switch that modulates macrophage polarisation and further disrupts energy balance. Myeloid-specific Kdm6a knockout in *Kdm6a*^*F/Y*^;*Lyz2-Cre* mice significantly reversed the high-fat diet (HFD)-induced M1–M2 imbalance in white adipose tissue (WAT) and blocked HFD-induced obesity. The brown adipose tissue (BAT) activity, WAT browning and energy expenditure were significantly increased in *Kdm6a*^*F/Y*^;*Lyz2-Cre* mice. Furthermore, Kdm6a regulated the Ire1α expression in a demethylase activity-dependent manner and augmented the M2 polarisation of macrophages. Macrophage with higher Kdm6a significantly promotes adipogenesis in white adipocyte and inhibits thermogenesis in beige adipocytes. These results suggest that the Kdm6a in macrophages drives obesity and metabolic syndrome by impairing BAT activity and WAT differentiation.

## Introduction

The trimethylation of histone H3 lysine 27 (H3K27me3) is a histone modification occurring on the amino-terminal tail of the core histone H3. This trimethylation is associated with the downregulation of nearby genes via the formation of heterochromatic regions [1]. H3K27me3 acts in opposition to trimethylation of histone H3 lysine 4 (H3K4me3), which activates gene expression [2]. Because of its dramatic and predictable effect on gene expression, H3K27me3 is the favourite marker of epigenetic researchers looking for inactive genes. H3K27me3 can be removed by histone lysine demethylases 6 family (Kdm6). The Kdm6 family contains a JmjC domain [3] and counters the enzymatic activity of polycomb repressive complex 2 [4] by removing di- and trimethyl groups from histone H3K27. The Kdm6 family includes Kdm6a (histone lysine demethylase 6a, also known as Utx) and Kdm6b (also known as Jmjd3), and has been shown to play important roles in a multitude of cellular processes, including differentiation [5, 6], senescence [7], somatic and germ cell reprogramming [8], cancer [9] and the inflammatory response [10]. The discovery of GSK-J4 provides an effective tool for the pharmacological inhibition of KDM6 members, which have been reported to inhibit the growth of TAL-1-positive T-ALL [11, 12], diffuse intrinsic pontine glioma [13, 14], melanoma [15] and neuroblastoma [16]. Most recently, the inhibition of Kdm6 family members with GSK-J4 was shown to abate nephropathy progression in diabetic db/db mice, suggesting that Kdm6a is also involved in the progression of metabolic diseases [17]. Type 2 diabetes and obesity induce similar transcriptional reprogramming of H3K27me3 in human myocytes [18]. Although Kdm6a is a target of the well-known antidiabetic drug metformin [19], the physiological role of Kdm6a in metabolic diseases, such as obesity and diabetes, is not fully understood. The relationship between Kdm6a and macrophage differentiation has not been investigated in the overfed state.

Obesity is associated with a state of chronic low-grade inflammation as active immune cells infiltrate adipose tissues [20]. Adipose tissue is composed of several types of tissue, including white, brown and beige fat, which play pivotal roles in metabolic homeostasis [21]. Various innate and adaptive immune cell types communicate with adipocytes and thereby maintain adipose function. In particular, adipose tissue macrophages (ATMs) are critical in orchestrating metabolic inflammation [22]. As the major effector cells mediating both adipose and systemic inflammation, ATMs respond to metabolic cues and are present in a spectrum of functionally distinct activation states, thereby exerting profound regulatory effects on metabolism. ATM M1 polarisation is thought to promote insulin resistance and type 2 diabetes [23], while ATM M2 polarisation, induced by eosinophil-derived type 2 cytokines (for example, the interleukins Il4), increases brown adipose tissue (BAT) activation and white adipose tissue (WAT) browning [24, 25]. ATM M2 polarisation thereby modulates adaptive thermogenesis and energy consumption through some cytokines’ (for example, the interleukins Il10) resistance [26]. Some genes have taken part in the polarisation of macrophage, such as Ire1α. Ire1α is an endoplasmic reticulum-resident transmembrane protein that senses ER stress. Myeloid-specific knockout of Ire1α has been reported to largely reversed high-fat diet (HFD)-induced M1–M2 imbalance in WAT and blocked HFD-induced obesity [23]. Downregulating Ire1α promoted M2 macrophage polarisation [27]. Emerging lines of evidence have revealed that Ire1α functions as a multifunctional signal transducer that responds to metabolic cues and nutrient stress conditions, exerting profound and broad effects on metabolic homeostasis [28]. But the relationship between Kdm6a and Ire1α expression is still limited.

In the present study, we tested the possibility that during chronic handling of excess nutrients, Kdm6a mechanistically converges with metabolic inflammation through Ire1α in macrophages, thereby initiating adipose dysfunction and causing the dysregulation of glucose and energy metabolism.

## Results

### Myeloid Kdm6a ablation prevents high fat diet-induced obesity

**Fig. 1:**
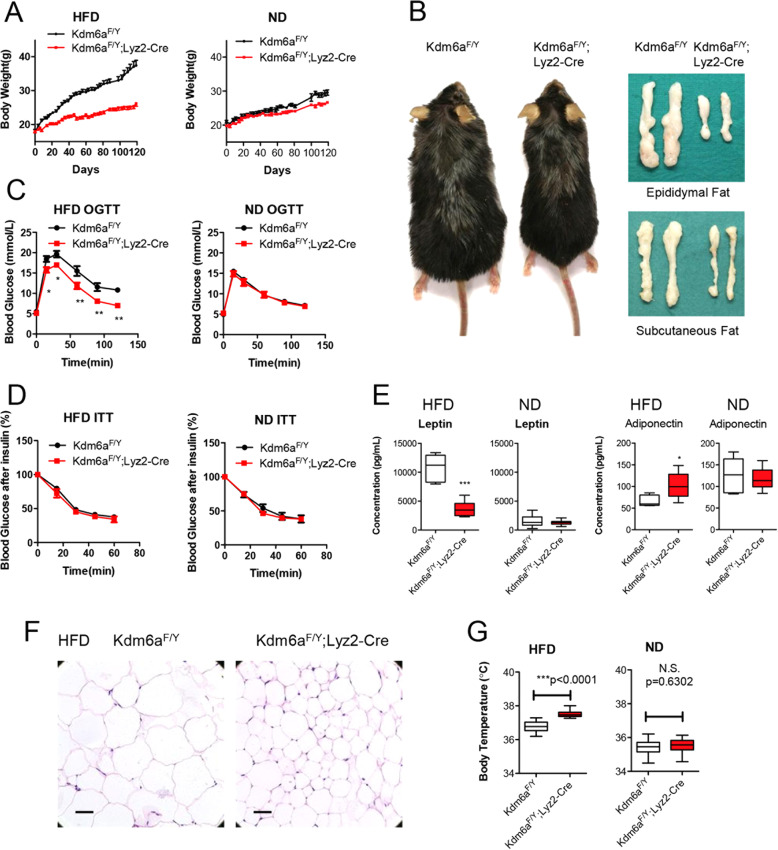
The myeloid-specific knockout of *Kdm6a* blocks high fat diet-induced obesity. **A** The body weight after the *Kdm6a*^*F/Y*^ mice and *Kdm6a*^*F/Y*^;*Lyz2*-*Cre* mice were fed a high fat diet (HFD) or normal diet (ND) for 16 weeks (*n* = 10 in each group). ***p < 0.001; **p < 0.005; *p < 0.05. N.S., not significant. **B** Representative images of HFD-fed wild-type and myeloid-specific knockout mice. The epididymal and subcutaneous adipose tissues are also presented. **C** The OGTT assay was used to detect blood glucose homeostasis in HFD- and ND-fed mice. **D** The ITT assay was used to detect blood glucose homeostasis in HFD- and ND-fed mice. **E** Serum leptin and adiponectin concentrations of mice fed with HFD or ND. **F** Representative images of H&E-stained WAT in the HFD-fed mice, bar = 100 μm. **G** The body temperature was recorded.

We first examined whether Kdm6a is involved in HFD-induced obesity (DIO) and metabolic inflammation in obese mice. We crossed floxed *Kdm6a*^*F/Y*^ mice with lysozyme 2-*Cre* (*Lyz2-Cre*) mice, in which Kdm6a protein expression was abolished in bone marrow-derived macrophages (BMDMs) and peritoneal macrophages but present in other examined tissues (Supplementary Fig. 1A, B). In comparison to their *Kdm6a*^*F/Y*^ counterparts, *Kdm6a*^*F/Y*^;*Lyz2-Cre* mice showed no defective developmental phenotypes when they were fed a normal chow diet (ND) (Fig. 1A). However, the *Kdm6a*^*F/Y*^;*Lyz2-Cre* mice were completely resistant to DIO, exhibiting significantly reduced body weight and adiposity (Fig. 1A, B). The blood glucose control in HFD-fed *Kdm6a*^*F/Y*^;*Lyz2*-*Cre* mice was significantly improved compared to that in the HFD-fed *Kdm6a*^*F/Y*^ mice (Fig. 1C). The HFD-fed *Kdm6a*^*F/Y*^;*Lyz2-Cre* mice showed markedly increased glucose tolerance, but the sensitivity to insulin was not influenced (Fig. 1D). In addition, the *Kdm6a*^*F/Y*^;*Lyz2*-*Cre* mice did not exhibit hyperleptinemia and hypoadiponectinemia when fed a HFD (Fig. 1E). Consistent with lower body weight and adiposity, the HFD-fed *Kdm6a*^*F/Y*^;*Lyz2-Cre* mice also showed an epididymal WAT (epWAT) phenotype with smaller adipocytes than their counterparts (Fig. 1F). The body temperature of *Kdm6a*^*F/Y*^;*Lyz2*-*Cre* mice in the HFD group was markedly higher than that of their *Kdm6a*^*F/Y*^ counterparts or the ND-fed mice (Fig. 1G). These data suggest that Kdm6a in myeloid cells couples overnutrition to the development of obesity and obesity-associated metabolic deterioration.

### Myeloid-specific Kdm6a knockout increases energy expenditure

**Fig. 2:**
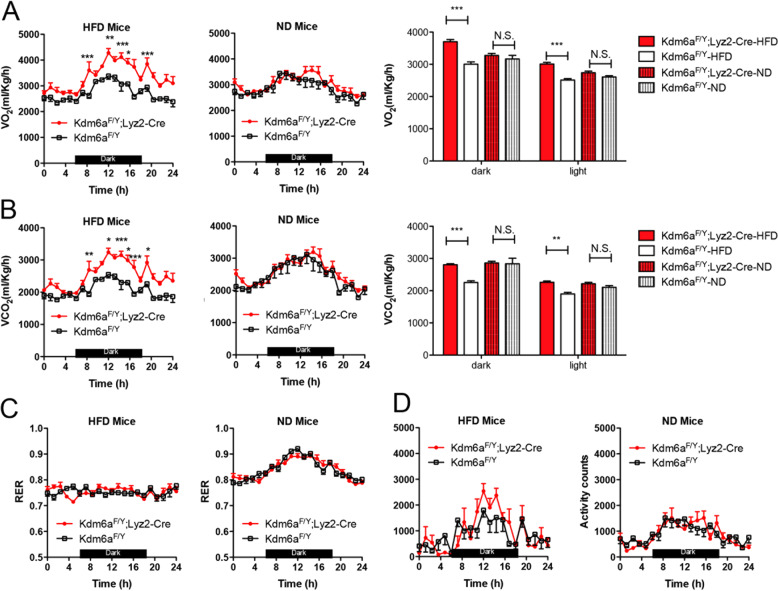
The myeloid Kdm6a ablation increases energy expenditure of DIO mice but not lean mice. **A** The mice were fed with HFD or ND for 16 weeks. The oxygen consumption (VO2) monitored over a 24-h period is shown as averaged values (*n* = 8 in each group). **B** Carbon dioxide production (VCO2) monitored over a 24-h period, shown as averaged values. **C** The RER was analysed for 24 h. **D** The locomotor activity were measured.

As the body temperature of the *Kdm6a*^*F/Y*^;*Lyz2*-*Cre* mice was significantly higher than that of the *Kdm6a*^*F/Y*^ mice when the mice were fed a HFD, we next performed an examination with a metabolic cage to determine whether the myeloid-specific Kdm6a knockout might be attributable to the increased energy consumption. The *Kdm6a*^*F/Y*^;*Lyz2*-*Cre* mice consumed a much higher volume of O_2_ (VO2) than their counterparts (Fig. 2A). There were no differences in VO2 consumption between the *Kdm6a*^*F/Y*^;*Lyz2*-*Cre* mice and the *Kdm6a*^*F/Y*^ mice on the ND-fed, and the *Kdm6a*^*F/Y*^;*Lyz2*-*Cre* mice mainly consumed O_2_ in the dark phase. Similarly, the Kdm6a-deficient mice produced a greater volume of CO_2_ (VCO2) than the *Kdm6a*^*F/Y*^ mice (Fig. 2B). There was no difference in the respiratory exchange rate (RER) between *Kdm6a*^*F/Y*^;*Lyz2*-*Cre* mice and *Kdm6a*^*F/Y*^ mice (Fig. 2C). The *Kdm6a*^*F/Y*^;*Lyz2*-*Cre* mice fed with the HFD showed no significant change of locomotor activity compared to the HFD-fed *Kdm6a*^*F/Y*^ mice or the ND-fed mice (Fig. 2D). These data suggest that the myeloid-specific Kdm6a knockout only caused increased energy consumption of HFD-fed mice.

### Kdm6a deficiency reverses the ATM M1–M2 imbalance

**Fig. 3:**
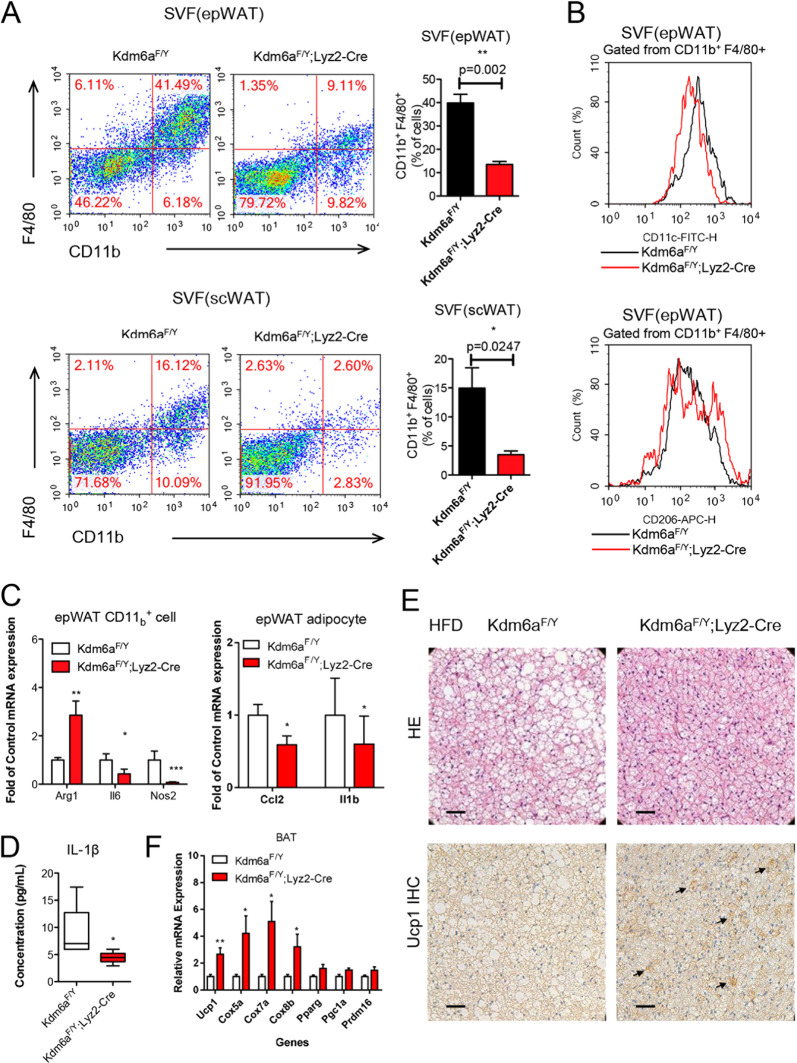
Kdm6a deficiency reverses the ATM M1–M2 imbalance. **A** Representative flow cytometry histograms and the quantitative data from the analysis of CD11_b_^+^ F4/80^+^ cells in SVFs of the epWAT and scWAT. **B** The percentage of CD11_c_^+^ cells gated from the CD11_b_^+^ F4/80^+^ population in epWAT was decreased in the *Kdm6a*^*F/Y*^;*Lyz2*-*Cre* mice. The percentage of CD206^+^ cells gated from CD11_b_^+^ F4/80^+^ population in epWAT was increased in the *Kdm6a*^*F/Y*^;*Lyz2*-*Cre* mice. **C** Realtime PCR assays to detect the abundance of the indicated mRNAs in the CD11_b_^+^ cells and adipocytes isolated from the epWAT. **D** The serum concentration of Il1β in the DIO mice. **E** The H&E-stained sections of HFD-fed mice indicating lipid droplets in the BAT. Arrows indicate the focal enhancement of Ucp1 expression in the BAT, bar = 100 μm. **F** Realtime PCR assays to detect the abundance of the indicated mRNAs in the BAT.

In order to examine the polarisation of macrophages in the adipose, we collected the adipose tissue and enriched the F4/80^+^ CD11_b_^+^ macrophages for further examination. The HFD-fed *Kdm6a*^*F/Y*^;*Lyz2-Cre* mice showed a phenotype with smaller adipocytes than their counterparts in the epWAT and subcutaneous WAT (scWAT). Flow cytometry analysis showed a significant ~34% and ~25% decrease in the number of F4/80^+^ CD11_b_^+^ cells in the epWAT and scWAT, respectively (Fig. 3A). In the epWAT of *Kdm6a*^*F/Y*^;*Lyz2*-*Cre* mice, the percentage of CD11_c_^+^ cells (M1 phenotype) gated from CD11_b_^+^ F4/80^+^ cells was lower than that in the epWAT of *Kdm6a*^*F/Y*^ mice, while the percentage of CD206^+^ cells (M2 phenotype) was much higher (Fig. 3B). We further collected the stromal vascular fraction (SVF) of the epWAT, enriched the CD11_b_^+^ cells and performed real-time PCR. Compared to the *Kdm6a*^*F/Y*^ CD11_b_^+^ cells, the CD11_b_^+^ cells of *Kdm6a*^*F/Y*^;*Lyz2*-*Cre* mice expressed much more M2 markers *Arg1* mRNA and less M1 markers *Nos2* and *Il6* mRNA (Fig. 3C). Furthermore, the expressions of *Ccl2* and *Il1β* mRNA were decreased in the adipocytes (Fig. 3C), which was consistent with the serum concentrations Il1β in the respective mice (Fig. 3D). To further examine the energy deposition and consumption, we dissected the BAT from mice, as BAT is the major organ that dissipates energy through the activation of mitochondrial uncoupling protein 1 (Ucp1) during adaptive thermogenesis. In the BAT of *Kdm6a*^*F/Y*^;*Lyz2*-*Cre* mice fed with the HFD, there were much lower lipid-droplet levels but higher focal enhancement of expression of Ucp1 (Fig. 3E). The higher expression of *Ucp1* mRNA was also observed in the BAT of *Kdm6a*^*F/Y*^;*Lyz2*-*Cre* mice (Fig. 3F), while there was no significant difference in mice fed with ND (Supplementary Fig. 1C). Other genes involved in mitochondrial oxidative phosphorylation, including *Cox5a*, *Cox7a* and *Cox8b*, were also increased in the BAT of *Kdm6a*^*F/Y*^;*Lyz2*-*Cre* mice at the mRNA level (Fig. 3F). The *Kdm6a*^*F/Y*^;*Lyz2*-*Cre* mice had significantly elevated expression of key thermogenic genes in the BAT, thus suggesting that these mice had increased BAT thermogenic capacity.

### Myeloid-specific Kdm6a knockout improves adaptive thermogenesis in mice

**Fig. 4:**
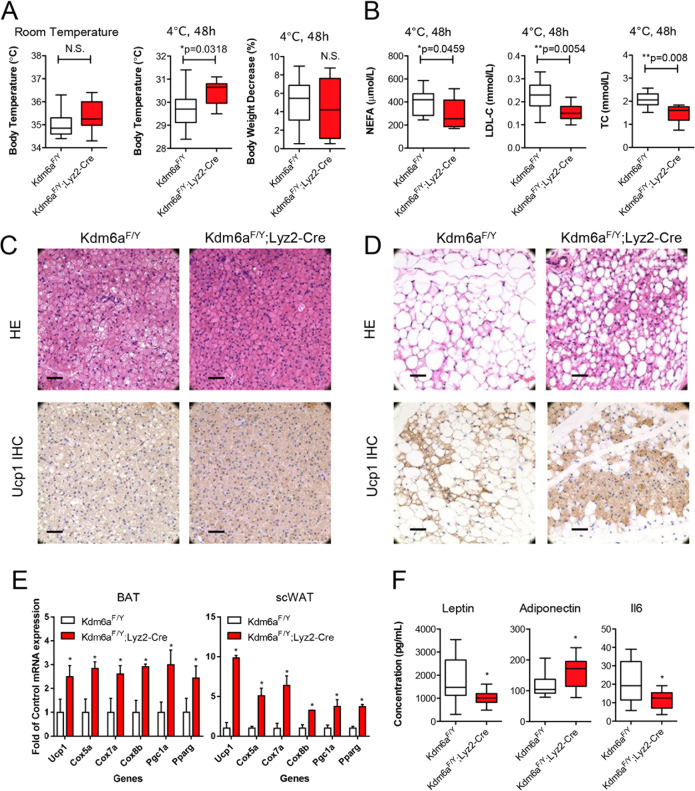
Myeloid Kdm6a ablation improves adaptive thermogenesis. **A** Body temperature and body weight of mice at room temperature and cold exposure at 4 °C for 48 h. **B** The concentration of nonesterified fatty acid (NEFA), low-density lipoprotein-cholesterol (LDL-C) and total cholesterol (TC) in the mouse serum after 48 h cold exposure. **C** H&E staining and IHC images of BAT in the *Kdm6a*^*F/Y*^ and *Kdm6a*^*F/Y*^;*Lyz2*-*Cre* mice, bar = 100 μm. **D** H&E staining and IHC images of scWAT in the mice, bar = 100 μm. **E** Realtime PCR assays to detect the abundance of the indicated mRNAs in the BAT and scWAT. **F** The serum concentrations of leptin, adiponectin and Il6 were measured.

Given the critical role of inducible beige adipocytes in the adaptive thermogenesis and the maintenance of energy balance, we also examined the effect of myeloid-specific Kdm6a knockout on adaptive thermogenesis in mice exposed to cold stress (4 °C). The *Kdm6a*^*F/Y*^;*Lyz2-Cre* mice maintained their body temperature at higher levels than the *Kdm6a*^*F/Y*^ mice after cold exposure for 48 h, but indicated no significant change in body weight (Fig. 4A). There was a significant difference between the groups in the concentration of nonesterified fatty acid (NEFA), low-density lipoprotein-cholesterol (LDL-C) and total cholesterol (TC) in the mouse serum after 48 h of cold exposure (Fig. 4B). However, cold stress decreased BAT lipid-droplet levels and increased Ucp1 expression to a greater extent in *Kdm6a*^*F/Y*^;*Lyz2*-*Cre* mice than control mice, thus indicating increased BAT activity (Fig. 4C). In addition, scWAT in *Kdm6a*^*F/Y*^;*Lyz2*-*Cre* mice subjected to cold exposure also exhibited marked remodelling (Fig. 4D), as evidenced by both increased induction of multilocular Ucp1-expressing beige adipocytes and the robust augmentation of cold-induced Ucp1 expression (Fig. 4E). Compared to the *Kdm6a*^*F/Y*^ mice, the *Kdm6a*^*F/Y*^;*Lyz2*-*Cre* mice exhibited lower concentrations of serum leptin and higher concentrations of adiponectin (Fig. 4F). Taken together, these data suggest that the Kdm6a deficiency in myeloid cells increases energy expenditure by promoting the thermogenic activity of both brown and beige adipose tissue.

### Kdm6a deficiency augments the polarisation of macrophages via demethylase activity

**Fig. 5:**
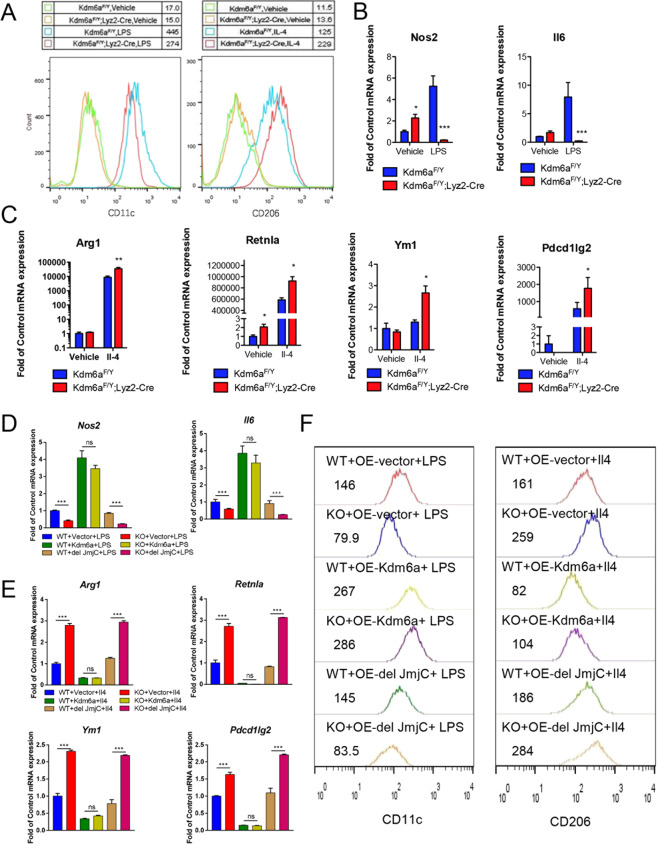
Kdm6a enhances the polarisation of macrophages via demethylase activity. **A** Representative flow cytometry histograms from the analysis of CD11_c_^+^ LPS-stimulated BMDMs and CD206^+^ Il4-stimulated BMDMs. **B** Realtime PCR assays to detect the abundance of the indicated mRNAs in LPS-stimulated BMDMs. **C** Realtime PCR assays to detect the abundance of the indicated mRNAs in the Il4-stimulated BMDMs. **D** Realtime PCR assays to detect the abundance of the indicated mRNAs in the LPS-stimulated upon ectopic expression of the full-length Kdm6a(Kdm6a) and Kdm6a truncation lacking the JmjC domain (del JmjC) in wild-type (WT) or Kdm6a-deficient (KO) BMDMs. **E** Realtime PCR assays to detect the abundance of the indicated mRNAs in the Il4-stimulated upon ectopic expression of the full-length Kdm6a (Kdm6a) and Kdm6a truncation lacking the JmjC domain (del JmjC) in wild-type (WT) or Kdm6a-deficient (KO) BMDMs. **F** Representative flow cytometry histograms from the analysis of CD11_c_^+^ LPS-stimulated BMDMs and CD206^+^ Il4-stimulated BMDMs. The median numbers of histogram were indicated.

Next, we considered whether macrophage Kdm6a might be involved in M1–M2 polarisation in a cell-autonomous manner. We used lipopolysaccharide (LPS) or Il4 stimulation of mouse BMDMs, which are in vitro models to mimic M1 and M2 polarisation, respectively. The LPS or Il4 cannot change the expression level of Kdm6a (Supplementary Fig. 1D, E). The flow cytometry analysis showed a significant decrease in LPS-stimulated CD11_c_^+^ and an increase in Il4-stimulated CD206^+^ cells gated from F4/80^+^ CD11_b_^+^ Kdm6a-deficient BMDMs (Fig. 5A). In the *Kdm6a*^*F/Y*^;*Lyz2-Cre* BMDMs, compared with the control *Kdm6a*^*F/Y*^ BMDMs, LPS increased *Nos2* and *Il6* mRNA expression to a lesser extent (Fig. 5B). In contrast, Il4-induced *Arg1*, *Ym1*, *Retnla* and *Pdcd1lg2* mRNA expressions were significantly elevated in *Kdm6a*^*F/Y*^;*Lyz2-Cre* BMDMs compared with control *Kdm6a*^*F/Y*^ BMDMs (Fig. 5C). These results indicate that loss of *Kdm6a* is sufficient to suppress LPS-induced M1 polarisation and promote Il4-induced M2 polarisation.

To examine whether the demethylase activity of Kdm6a is involved in modulating the polarisation of macrophages, we constructed a full-length Kdm6a-expressing plasmid, and a Kdm6a truncation lacking the JmjC domain (Kdm6a del JmjC). The overexpression of the full-length Kdm6a significantly reduced the H3K27me3 levels, while the overexpression of the truncated Kdm6a did not change the H3K27me3 levels (Supplementary Fig. 1F). The re-expression of full-length Kdm6a significantly increased the expression of M1 markers, *Nos2* and *Il6* mRNA, in both types of BMDMs (Fig. 5D). Meanwhile, the re-expression of full-length Kdm6a inhibited the expression of M2 molecular markers, such as *Arg1*, *Ym1*, *Retnla* and *Pdcd1lg2* (Fig. 5E). However, the introduction of the JmjC-depleted Kdm6a could not increase the M1 markers or decreased the M2 markers as the full-length Kdm6a did (Fig. 5D, E). The data of flow cytometry also showed that the re-expression of full-length Kdm6a increased the number of CD11_c_-positive macrophages and decreased the CD206-positive macrophages, while the JmjC-depleted Kdm6a failed to change M1 or M2 markers as the full-length Kdm6a did (Fig. 5F). These data suggest that Kdm6a regulates the polarisation of macrophages through the presence of its functional JmjC domain.

### Kdm6a epigenetically reduces H3K27me3 modification at Ire1α loci to regulate macrophage polarisation

**Fig. 6:**
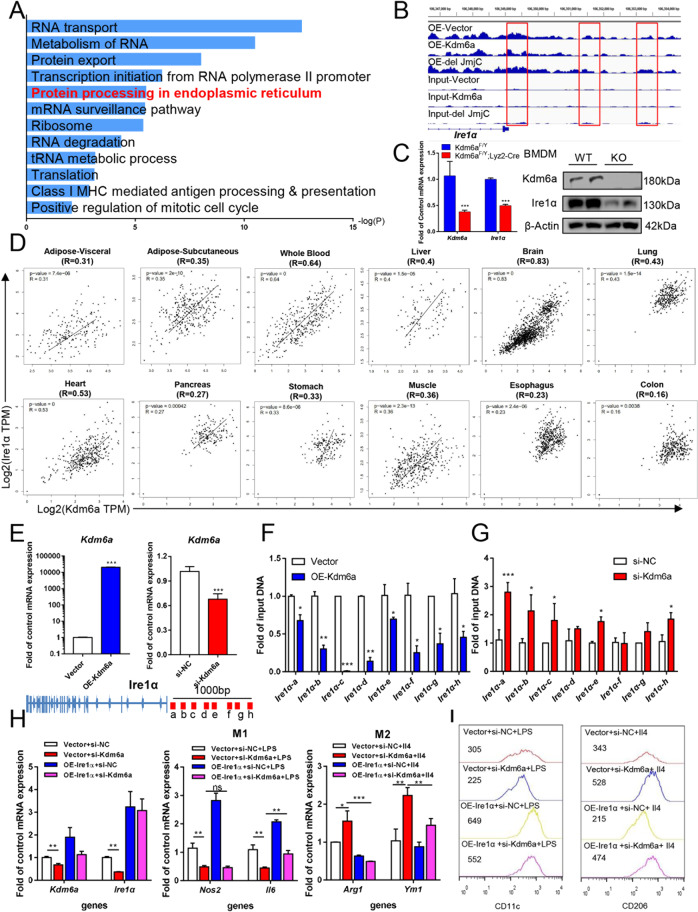
Kdm6a regulates M1–M2 polarisation through Ire1α expression. **A** The enrichment of KEGG pathway between OE-Kdm6a and OE-Vector BMDMs in the ChIP-Seq assay. **B** Genome browser view of normalised ChIP-Seq signals of H3K27me3 at the *Ire1α* locus in vector, OE-Kdm6a and OE-del JmjC BMDMs. **C** Realtime PCR assays and immunoblotting assays indicated Ire1α expression in Kdm6a-deficient BMDMs compared to the counterparts. **D** The expressions of *Kdm6a* and *Ire1α* mRNA are positively correlated in multiple human organs from the GTEx database. **E** Realtime PCR assays to detect the abundance of the indicated mRNAs in the Il4-stimulated BMDMs after overexpression of Kdm6a or knocking down with specific siRNA against Kdm6a. **F** ChIP-qPCR analysis of H3K27me3 modifications at the *Ire1α* loci upon ectopic expression of Kdm6a in BMDMs. **G** ChIP-qPCR analysis of H3K27me3 modifications at the *Ire1α* loci upon si-Kdm6a or si-NC treatment in BMDMs. **H** Realtime PCR assays to detect the abundance of indicated mRNAs in BMDMs. The cells were transfected with control group (vector + si-NC), Ire1α (OE-Ire1α + si-NC), siRNAs against Kdm6a (vector + si-Kdm6a) or Ire1α overexpression plus si-Kdm6a (OE-Ire1α + si-Kdm6a). ***p < 0.001; **p < 0.005; *p < 0.05. N.S., not significant. **I** Representative flow cytometry histograms to analyse the median number of LPS-stimulated CD11c^+^ BMDMs and Il4-stimulated CD206^+^ BMDMs.

To explore the way Kdm6a affects macrophage, we transfected the empty expressing vector, the full-length Kdm6a and the JmjC-depleted Kdm6a construct into BMDMs, then performed ChIP-Seq experiment with H3K27me3 antibodies. The enrichment of KEGG pathway indicates ‘protein processing in endoplasmic reticulum’ gained the high score in OE-Kdm6a BMDMs compared to the Vector group (Fig. 6A). From the genomic browse of normalised ChIP-Seq signals at the *Ire1α* locus, the peaks of H3K27me3 modifications in the OE-Kdm6a group are obviously lower than those of the Vector group, while there was no obvious difference between the OE-del JmjC group and the OE-Vector group (Fig. 6B). We did not find obvious H3K27me3 peaks at the loci of M2 markers, such as *Arg1*, *Retnla*, *Ym1 and Pdcd1lg2* (Supplementary Fig. 2A). The level of Ire1α decreased in Kdm6a-deficient BMDMs compared to the wild-type cells (Fig. 6C). In the GTEx database, we have found that the expressions of *Kdm6a* and *Ire1α* mRNA are significantly correlated in multiple tissues. The *R*-value of correlation in human whole blood is as high as 0.64 (Fig. 6D). To detect whether the Ire1α is the direct target gene silenced by H3K27me3 modification, we changed the expression of Kdm6a in BMDMs (Fig. 6E) and examined the H3K27me3 levels at the *Ire1α* loci with ChIP-qPCR analysis. The ectopic expression of Kdm6a in BMDMs decreased the H3K27me3 levels at promoter region of Ire1α loci (Fig. 6F), while knocking down Kdm6a with siRNAs significantly increased the H3K27me3 levels (Fig. 6G). To identify if the Kdm6a regulates the polarisation via Ire1α, the BMDMs were knocked down with specific siRNAs to Kdm6a and overexpressed with full-length Ire1α. The abundance of M1 and M2 marker mRNAs were detected in the LPS- or Il4-stimulated BMDMs, respectively. OE-Ire1α significantly reversed the si-Kdm6a decreased *Il6* mRNA and blocked the si-Kdm6a increased *Arg1* and *Ym1* mRNAs (Fig. 6H). In the flow cytometry analysis, the overexpression of Ire1α increased the count number of CD11_c_^+^ LPS-stimulated BMDMs and decreased the number of CD206^+^ Il4-stimulated BMDMs (Fig. 6I). These data suggest that Kdm6a regulates M2 polarisation through directly reduced H3K27me3 levels at the *Ire1α* loci and increased Ire1α expression.

### Kdm6a deficiency induces the Il10 signalling in macrophage and regulates adipocyte differentiation

**Fig. 7:**
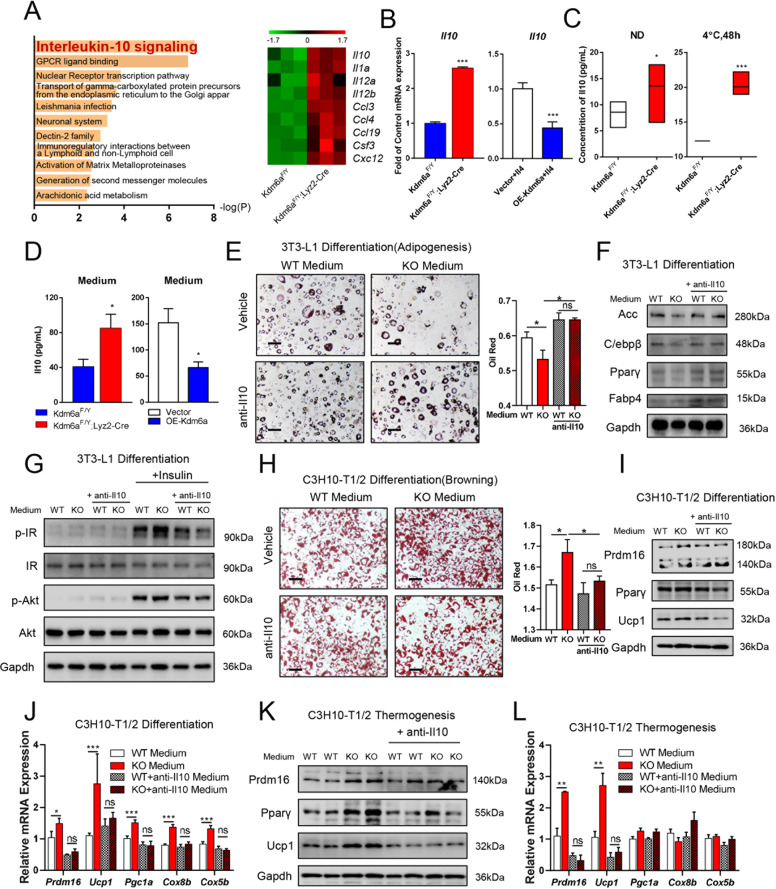
Kdm6a deficiency regulates differentiation of adipocyte through Il10. **A** The enrichment of KEGG pathway indicates the Il10 signalling gained highest score in Kdm6a-deficient BMDMs compared to the wild-type cells. **B** Realtime PCR assays to detect the abundance of *Il10* mRNA upon ectopic expression of Kdm6a or Kdm6a-deficient BMDMs. **C** Concentrations of Il10 in the serum of wild-type and Kdm6a-deficient mice fed with ND or after exposed to 4 °C for 48 h. **D** Concentrations of Il10 in culture medium of Kdm6a-deficient BMDMs or upon ectopic expression of Kdm6a. **E** The representative oil red staining images of 3T3-L1 differentiation, bar = 200 μm. **F** Immunoblotting assays to ACC, C/ebpβ, Pparγ and Fabp4 in wild-type or Kdm6a-deficient BMDMs medium-treated 3T3-L1 cells in the presence or absence of Il10 neutralising antibody. **G** Immunoblotting assays to phosphorylated insulin receptor and phosphorylated Akt levels in differentiated 3T3-L1 cells, which were incubated with indicated BMDMs medium. **H** The representative oil red images and quantitative data of differentiation in C3H10-T1/2 cells after received the indicated conditional medium from BMDMs, bar = 200 μm. **I** Immunoblotting assays to examine the Ucp1 and Prdm16 proteins in the C3H10-T1/2 cells. During the differentiation, cells were incubated with indicated BMDMs medium. **J** Relative mRNA abundance of differentiation markers was detected with Realtime PCR assay. During the differentiation, C3H10-T1/2 cells were incubated with indicated BMDMs medium. **K** Immunoblotting assays to indicate the thermogenesis markers in the differentiated C3H10-T1/2 cells, after the already differentiated cells treated with indicated BMDMs medium. **L** Relative mRNA abundance of the thermogenesis markers in the differentiated C3H10-T1/2 cells was detected with Realtime PCR, after the already differentiated cells were treated with conditional medium from wild-type or Kdm6a-deficient BMDMs.

To further identify the most changed signalling pathway by Kdm6a knockout, we performed RNA-Seq in wild-type and Kdm6a-knockout macrophages. The enrichment of KEGG pathway indicated that the Interleukin-10 signalling gained the highest score in Kdm6a-deficient BMDMs compared to the wild-type cells (Fig. 7A). The *Il10* mRNA in Kdm6a-deficient BMDMs significantly increased compared to the wild-type group, but decreased after Kdm6a were overexpressed in BMDMs (Fig. 7B). The serum Il10 levels of Kdm6a-deficient mice are significantly higher than those of wild-type mice, especially exposed to cold stress (Fig. 7C). We also collected the conditional medium from Kdm6a-deficient and wild-type BMDMs (Fig. 7D).

In order to examine the function of Il10 during the differentiation of white and brown adipocytes, we chose the concentration of 0, 25 and 50 pg/ml Il10 to incubate 3T3-L1 cells during the differentiation into white adipocytes (Supplementary Fig. 2B). Il10 dose-dependently decreased the differentiation of white adipocytes (Supplementary Fig. 3A), reduced the mRNA expression of *Fabp4* and *C/ebpβ* (Supplementary Fig. 3B) and inhibited the expression of ACC protein (Supplementary Fig. 3C). On the other hand, Il10 effectively promoted the differentiation of C3H10-T1/2 into brown adipocytes (Supplementary Figs. 2D and  3D), increased the mRNA levels of *Prdm16*, *Ucp1*, *Pgc1a*, *Cox8b* and *Cox5b* (Supplementary Fig. 3E), and upregulated the expression of Ucp1 and Prdm16 (Supplementary Fig. 3F). These data suggest that Il10 inhibits the differentiation of white adipocyte, while it promotes the differentiation of brown adipocyte.

To examine whether the presence of Kdm6a in BMDMs regulated the differentiation of white and brown adipocytes through Il10 production, we supplemented the neutralising antibody against mouse Il10 into the culture medium of BMDMs. As indicated in Fig. 7E and Supplementary Fig. 4A, the conditional medium from OE-Kdm6a BMDM increased the differentiation of the 3T3-L1 cells into white adipocytes and the conditional medium from Kdm6a-deficient BMDMs inhibited the differentiation, while the Il10 neutralising antibody blocked the change. The less-Kdm6a-expressed BMDM medium decreased the levels of ACC, C/ebpβ, Pparγ and Fabp4 during the differentiation of 3T3-L1 cells, which could be reversed by the Il10 neutralising antibody (Fig. 7F and Supplementary Fig. 4B). We also examined the role of Il10 in the conditional medium on the insulin sensitivity of already differentiated white adipocytes (Supplementary Fig. 2C). The less-Kdm6a-expressed BMDM medium promoted the insulin sensitivity of adipocytes, but the supplement of neutralising antibody of Il10 blocked the effect (Fig. 7G and Supplementary Fig. 4C). The less-Kdm6a-expressed BMDM medium significantly enhanced the differentiation of C3H10-T1/2 cells into brown adipocytes, while the differentiation was reversed by Il10 neutralising antibody (Fig. 7H and Supplementary Fig. 4D). Meanwhile, the less-Kdm6a-expressed BMDMs medium promoted the expression of Ucp1 and Prdm16 of C3H10-T1/2 cells, but the supplement of neutralising antibody of Il10 blocked the effect (Fig. 7I, J and Supplementary Fig. 4E, F). Moreover, the levels of Ucp1, Prdm16 and Pparγ in the already differentiated C3H10-T1/2 cells were also increased by the less-Kdm6a-expressed BMDM medium, while the Il10 neutralising antibody blocked the effect of Kdm6a-deficient BMDM medium (Supplementary Fig. 2E and Fig. 7K, L).

Taken together, these data suggest that the deficiency of Kdm6a in BMDMs regulates the differentiation of white adipocyte and enhances the differentiation and thermogenesis of brown adipocyte through increasing the production of Il10.

### Kdm6a deficiency in BMDM regulates differentiation of adipocyte depending on Ire1α

**Fig. 8:**
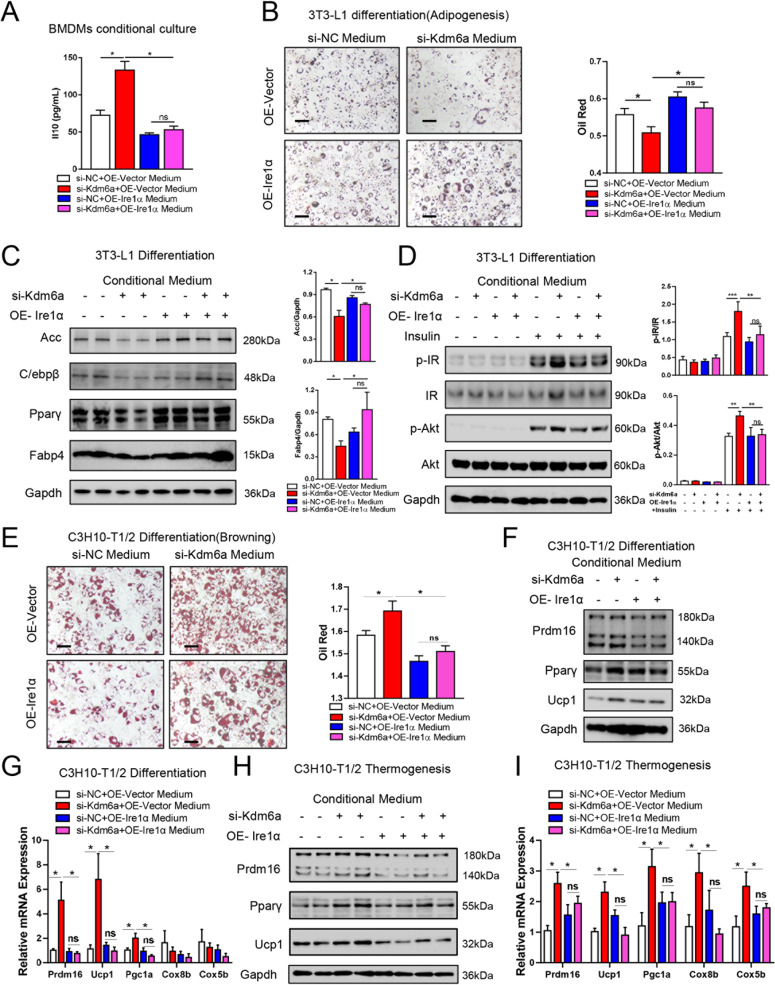
Kdm6a-deficient BMDM regulates differentiation of adipocyte via Ire1α. **A** Concentrations of Il10 in culture medium from indicated BMDMs. **B** The representative images of oil red staining of 3T3-L1 adipocytes and the quantitative data. During the differentiation, cells were treated with conditional medium from indicated BMDMs, bar = 200 μm. **C** Immunoblotting assays to detect ACC, C/ebpβ, Pparγ and Fabp4 proteins in 3T3-L1 cells after received the treatment of indicated BMDMs conditional medium. **D** Immunoblotting assays to examine the p-Insulin Receptor and p-Akt levels in 3T3-L1 cells in response to insulin incubation, after cells received the indicated BMDMs conditional medium. **E** The representative images of oil red staining of C3H10-T1/2 adipocytes and the quantitative data. During the differentiation, cells were treated with conditional medium from indicated BMDMs, bar = 200 μm. **F** Immunoblotting assays to examine the Ucp1 and Prdm16 proteins in the C3H10-T1/2 cells. During the differentiation, cells were incubated with conditional medium from indicated BMDMs. **G** Relative mRNA abundance of shown mRNAs in the C3H10-T1/2 cells. During the differentiation, cells were incubated with conditional medium from indicated BMDMs. **H** Immunoblotting assays to indicate the thermogenesis markers in the already differentiated C3H10-T1/2 cells, after the differentiated cells were treated with indicated BMDMs medium. **I** Relative mRNA abundance of thermogenesis markers in the differentiated C3H10-T1/2 cells was detected with Realtime PCR, after the differentiated cells were treated with indicated BMDMs medium.

Next, we knocked down the expression of Kdm6a plus overexpressing Ire1α in BMDMs and collected the culture medium of BMDMs to incubate 3T3-L1 or C3H10-T1/2 preadipocytes. As the shown data of ELISA assay, the si-Kdm6a plus overexpressing Ire1α BMDMs decreased the level of Il10 production compared to single si-Kdm6a group of BMDMs (Fig. 8A). The conditional medium from Ire1α-overexpressed BMDMs (OE-Ire1α BMDMs medium) significantly promoted 3T3-L1 to differentiate into white adipocyte. The medium from si-Kdm6a plus overexpressing Ire1α BMDMs promoted the differentiation of 3T3-L1 cells when compared to the effect of single si-Kdm6a group of BMDMs (Fig. 8B). The medium from si-Kdm6a plus overexpressing Ire1α BMDMs also restored the decreased levels of ACC, C/ebpβ, Pparγ and Fabp4 by single si-Kdm6a BMDMs medium during the differentiation of 3T3-L1 preadipocytes (Fig. 8C). Moreover, the medium from si-Kdm6a plus overexpressing Ire1α BMDMs reduced the insulin sensitivity of already differentiated adipocytes when compared to the effect of single si-Kdm6a BMDMs medium (Fig. 8D). On the other hand, the medium from si-Kdm6a plus OE-Ire1α-treated BMDMs more efficiently inhibited the differentiation of C3H10-T1/2 cells into brown adipocyte than the single si-Kdm6a group (Fig. 8E). Compared to the si-Kdm6a group, the medium from si-Kdm6a plus OE-Ire1α treated BMDMs significantly decreased the expression of Ucp1 and Prdm16 during the differentiation of C3H10-T1/2 cells (Fig. 8F, G). When the differentiated C3H10-T1/2 cells were incubated with si-Kdm6a plus OE-Ire1α BMDMs medium, the levels of Ucp1, Prdm16 and Pparγ were also lower than the single si-Kdm6a group (Fig. 8H, I). These data indicate that Kdm6a deficiency regulates the phenotype of macrophages by decreasing the expression of Ire1α, leading to changes in the production of Il10, thus affecting white and brown adipocytes.

## Discussion

Kdm6a is a key histone demethylase that reactivates the expression of target genes by decreasing the H3K27me3 modification. Histone methylation has been rarely linked to metabolic pathways or obesity. Our findings connected epigenetic regulation with metabolism, presenting new insight to define the function of Kdm6a in modulating energy storage and expenditure.

We observed that the expression levels of Kdm6a and Ire1α changed in the same direction. When we overexpressed or knocked out Kdm6a in BMDMs, the expression of Ire1α increased or decreased, and vice versa. These data indicate that there may be a feed-forward mechanism of the regulation between Ire1α and Kdm6a. It has been reported that the presence of Ire1α in BMDMs changes the phenotype of macrophages and affects the obese status of mice [23, 27].Therefore, the feed-forward mechanism between Ire1α and Kdm6a will possibly strengthen their expression and function to regulate the phenotype of macrophages.

Ire1α has been reported to regulate M2 polarisation, previously [23, 27]. In the present study, we found that Kdm6a directly decreased the H3K27me3 level at the promoter region of *Ire1α* loci, and thereby resulted in upregulating the expression of *Ire1α* mRNA. Our results indicate that Kdm6a-deficiency decreased the Ire1α expression and upregulated M2 macrophage marker Arg1 and Ym1 expression. Our in vitro and in vivo experiments showed that the loss of Kdm6a function in macrophages reverses the M1–M2 imbalance and promotes the secretion of Il10. It has been reported that Il10 plays a potent role in adipocyte differentiation and insulin resistance [26]. Consistently, the differentiation of white adipose was increased and thermogenesis of brown adipose was decreased by the supernatant of Kdm6a-overexpressed macrophage. Among the enriched pathways of significantly different peaks between the Kdm6a and Vector groups, we also find RNA transport and metabolism were included. We only detected the function of Ire1α during the Kdm6a-induced polarisation in this study. Further efforts should be performed to identify more target genes of Kdm6a in regulating the function of macrophages.

Our in vivo data also indicate that the weight gain rate of *Kdm6a*^*F/Y*^ mice is faster than that of *Kdm6a*^*F/Y*^;*Lyz2-Cre* mice with HFD. In order to study whether Kdm6a presence in macrophage is affecting the adipose differentiation, we carried out a cold stimulation experiment. After 48 h of cold exposure, the body temperature of the *Kdm6a*^*F/Y*^;*Lyz2-Cre* mice was significantly higher than that of the *Kdm6a*^*F/Y*^ mice, and their browning or beige adipose tissue were much higher than those of their counterparts. These findings are consistent with the hypothesis that the loss of Kdm6a function in macrophages promotes the M2 polarisation, leading to WAT browning and BAT activation. It has been reported that immune cells within the adipose tissue niche closely interact with adipocytes to regulate local and systemic metabolic homeostasis [20]. We also performed the in vitro study to identify the function of Kdm6a in macrophage to promote the white adipocyte to adipogenesis, while the culture medium of Kdm6a-overexpressed macrophage inhibited the expression of thermogenesis genes, such as Ucp1 and Prdm16. However, there could be other potential mechanisms beyond control of WAT/BAT function by Kdm6a in macrophages and further study should be performed. Kdm6b (also known as Jmjd3) has also been reported to regulate the M2 polarisation of macorphages [29]. As the catalytic domains of the two demethylases are similar, they both reduced the H3K27me3 modification. However, we did not examine the role of Kdm6b in the macrophage of obese patients or mouse models in this study. Further efforts should be performed to confirm the potential role of Kdm6b in the obesity.

In conclusion, Kdm6a in macrophages drives obesity and metabolic syndrome by impairing BAT activity and WAT differentiation. Our findings point to a new mechanism through which the epigenetic modulation of histone modification controls adaptations to thermogenic activation.

## Materials and methods

### Primary culture of mouse BMDM

The femurs and tibias of the hind limbs of 10-week-old male mice were isolated, and the bone marrow was removed with a sterile syringe. After the red blood cells were lysed with red blood cell lysis buffer, the sediment was resuspended and cultured in MEMα medium containing 10 ng/ml macrophage colony-stimulating factor (M-CSF, Peprotech, 300-25) for 6 days and then cultured in MEMα medium without M-CSF for 3 days to obtain the BMDMs. Notably, on the 4th day of culturing, MEMα containing M-CSF (10 ng/ml) was added for nutritional supplementation. Finally, to polarise the BMDMs to M1 or M2 phenotypes, the cells were cultured in a medium containing 100 ng/ml LPS (Multi Science, CS0006) or 20 ng/ml Il4 (Peprotech, 214-14) for 24 h.

### Chemical preparation

Insulin was purchased from Novo Nordisk (Novolin R), and Il10 was purchased from R&D Systems (217-IL-010). Neutralising antibody against Il10 were purchased from BioXcell (BE0049).

### Animals

The *Kdm6a*^*F/Y*^ (Jackson Laboratory stock number 024177) [30] and *Lyz2-Cre* (Jackson Laboratory stock number 004781) [31] mouse strains have been described previously. The mice were backcrossed with wild-type C57BL/6 J mice for at least 10 generations. The generation of *Kdm6a*^*F/Y*^;*Lyz2-Cre* mice was achieved by successive breeding. The primers used for genotyping the mice are listed in Supplementary Table 2.

Animal experiments were performed in accordance with the relevant ethical regulations of the Shanghai Ninth People’s Hospital. The study was approved by the Animal Experimental Ethics Committee of Shanghai Ninth People’s Hospital, affiliated with the Shanghai Jiao Tong University School of Medicine. Six-week-old male mice were housed in a temperature-controlled (22 °C) room with a 12-h light/dark cycle and given free access to food and water. Mice were fed either a HFD (60 kcal% fat content; Research Diets Formula D12331; Research Diets, Inc., New Brunswick, NJ) or a standard chow diet (ND; 11 kcal% fat content; Research Diets Formula D12329; Research Diets, Inc.) for 16 weeks. The body weight was recorded weekly throughout the study. The mice were sacrificed by cervical dislocation. Tissues were collected from each mouse, snap frozen in liquid nitrogen and stored at −80 °C.

For the cold exposure experiment, mice were single-caged and housed at 4 °C for 48 h in the auto artificial climate cabinet (GDN-260A-4, Ningbo le electrical instrument, China). Animals were exposed to a standard 12 h:12 h light:dark cycle and had free access to food pellets and water. Rectal temperatures were recorded at the end of cold exposure.

### Examination on indirect calorimetry and locomotor activity

Locomotor activity, oxygen consumption and carbon dioxide production were simultaneously measured in individually housed mice with a LabMaster system (TSE Systems). Mice were acclimatised for 2 days, and data were collected for 2 days and analysed. The light and feeding conditions were consistent with those in the home cages.

### OGTT and ITT

For the oral glucose tolerance test (OGTT), the mice were fasted for 15 h, and glucose (1 g/kg for lean mice and mice with DIO) was administered per os (P.O.). Blood glucose levels were measured from the tail before oral administration and 15, 30, 60, 90 and 120 min after administration.

For the insulin tolerance test (ITT), mice were fasted for 6 h, starting at 8 a.m. and lasting until 2 p.m. Recombinant human insulin (1 IU/kg for lean mice and mice with DIO) was administered by i.p. injection. Blood glucose levels were measured from the tail before insulin administration and 15, 30, 45 and 60 min after administration.

### Hormone and metabolite measurements in mouse serum

We utilised the corresponding ELISA or assay kits according to the manufacturers’ instructions to measure plasma leptin (R&D), Il10 (Multisciences), adiponectin (Millipore), cholesterol (Roche) and triglycerides (Roche). We used 5 μl of serum sample from lean mice and 5 μl of 5× diluted serum sample from mice with DIO for the leptin ELISA. We used 5 μl of serum from lean mice, mice with DIO for the insulin ELISA, 3 μl for the cholesterol assay and 3 μl for the triglyceride assay.

### H&E staining

At the end of the treatment period, we dissected the adipose and stored them in 10% buffered formalin phosphate. Paraffin-embedded sections were haematoxylin and eosin (H&E) stained as per the standard procedures.

### Separation of macrophages and stromal vascular fractions from adipose tissue

We added PBS to the subcutaneous and epididymal fat tissues of the mice, cut them into pieces and added 0.1% collagenase. After shaking for 45 min, digested tissues were filtered through a 70-µm nylon mesh and centrifuged at 500 × *g* for 5 min. Then, we lysed the red blood cells with lysis buffer and terminated the reaction with PBS.

### Flow cytometry analysis

After adding the desired combination of fluorochrome-conjugated antibodies (M1 flow dyes included PE-anti-F4/80, FITC-anti-CD11b and APC-anti-CD11c; M2 flow dyes included PE-anti-F4/80, FITC-anti-CD11b and APC-anti-CD206), the cells to be tested were incubated on ice for 15 min in the dark. Thereafter, the cells were subjected to flow cytometry analysis. Data were analysed with FlowJo Software. Antibody were listed in Supplementary Table 1.

### Nucleic acid transfection

Ribobio Company, China, synthesised small RNA fragment for mouse Kdm6a (UUAAAUAGCAUUUAAUAGCAU) and one negative control (a scrambled sequence). Ire1α, full-length Kdm6a and del JmjC main of Kdm6a were separately constructed into pCMV3 vectors. Cells were transfected with different Nucleic acid fragment or expression vectors using Lipofectamine 3000 (Life Technologies).

The siRNA transfection was conducted at a cell density of about 60%. First, the cells were starved with serum-free medium for 4 h. siRNA (transfection final concentration: 200 nM) and Lipo3000 were diluted to OPTI-MEM, respectively, and then the transfection was mixed and left for 5 min at room temperature. They were then mixed and placed for 8 min before being added to the cells, and changed to serum-containing medium after 4 h.

### Immunoblotting analysis

The cells were lysed in lysis solution, and the proteins were separated on SDS-PAGE gels and transferred to PVDF membranes. Next, 5% milk powder-containing buffer was used to reduce the nonspecific background. Bands were detected using various antibodies, as indicated. The membranes were incubated with primary antibodies at 4 °C overnight and secondary antibodies for 1 h at room temperature before exposure to an AI600 system in the dark for band detection. The catalogue numbers of the primary antibodies are listed in Supplementary Table 1.

### Immunohistochemistry

For immunohistochemistry (IHC), adipose tissue sections were rehydrated with xylene solution and different concentrations of anhydrous ethanol solution in turn, then boiled for 10 min at 95–100 °C in sodium citrate antigen retrieval solution (C1032, Solarbio) for antigen retrieval, and endogenous peroxidases were quenched with 3% hydrogen peroxide. The sections were incubated with anti-Ucp1 overnight at 4 °C, followed by incubation with an HRP-conjugated secondary antibody (GK500705, Gene Tech). The DAB staining kit was used according to the instructions from the manufacturer. After being counterstained with haematoxylin, sections were dehydrated with different concentrations of anhydrous ethanol solution and xylene solution in turn and then sealed with gum (BL704A, Life Science). Microscopy analysis was performed with a ZEISS AXIO BX61 microscope.

### Reverse-transcription polymerase chain reaction and quantitative PCR assays

Quantitative PCR was performed using an ABI Prism 7300 system (Applied Biosystems, Foster City, CA, USA) and SYBR Green (Takara, Dalian, China). For PCR, up to 1 μl of cDNA was used as a template. The thermal cycling conditions were 95 °C for 10 s followed by 40 cycles of 95 °C for 5 s and 60 °C for 30 s. A primer efficiency of >90% was confirmed with a standard curve spanning four orders of magnitude. Following the reactions, the raw data were exported using 7300 System Software 4 v1.3.0 (Applied Biosystems) and analysed. The primers used are listed in Supplementary Table 2.

### Adipocyte differentiation

For the white adipocyte differentiation, 3T3-L1 cells were treated for 8 days with differentiation medium including insulin, dexamethasone and 3-isobutyl-1-methylxanthine (IBMX) at concentrations of 1 μg/ml, 0.25 μM and 0.5 mM, respectively. The supernatant medium of macrophage was added to 3T3-L1 cells according to the experimental requirements.

For the brown adipocyte differentiation, the C3H10-T1/2 cells were first incubated with culture medium of macrophage for 2 days, followed by replacement with differentiation-inducing reagents including IBMX (0.5 mM), indomethacin (125 nM), dexamethasone (1 μM), triiodothyronine (1 nM), insulin (850 nM) and rosiglitazone (1 μM) for 2 days. Then, the medium was changed with differentiation maintenance medium containing T3 (1 nM), insulin (850 nM) and rosiglitazone (1 μM). The maintenance medium was changed every 2 days, mRNA and protein levels were detected after differentiation and maturation on the 8th day.

### Oil Red staining

After being fixed with 4% paraformaldehyde at room temperature for 30 min, the cells were stained with the newly prepared Oil Red working fluid for 1 h. Then, the cell plates were observed under the microscope and photographed. In addition, the cell plates were dissolved with isopropanol for 15 min, and the absorbance value of 520–630 nm was determined.

### Chromatin Immunoprecipitation (ChIP) and ChIP-seq assay

BMDMs were knocked down with specific siRNAs or overexpressed with full-length Kdm6a for 48 h. ChIP assays were performed using an EZ ChIP kit (Cell Signaling Technology, 9003). Briefly, BMDMs (1 × 10^7^ cells) were fixed with 1% formaldehyde and then neutralised by adding 0.125 M glycine. Cells were collected and lysed in cell lysis buffer containing SDS and a cocktail of protease inhibitors. The lysates were sonicated to obtain soluble chromatin with an average length of 500 bp. After a 1:10 dilution in dilution buffer, the chromatin solutions were precleared and incubated with anti-H3K27me3 antibodies. Next, the mixtures were incubated overnight at 4 °C on a rotating platform. The immunocomplexes were captured by protein A/G-Sepharose beads. After extensive washing, the bound DNA fragments were eluted, and the resulting DNA was subjected to real-time PCR analysis using the ChIP primer. ChIP-seq assay were performed in the Genewiz, China.

### RNA-Seq analysis

We performed RNA-seq analysis using the NovelBrain Cloud Analysis Platform, China. In brief, total RNA was extracted using TRIzol reagent (Invitrogen) according to the manufacturer’s protocol for the indicated cell samples. The cDNA libraries were then constructed for each pooled RNA sample using the VAHTSTM Total RNA-seq (H/M/R). Differential gene and transcript expression levels from the RNA sequences were examined using TopHat and Cufflinks. The gene expression level was determined by the FPKM method. We applied the DESeq algorithm to screen out the differentially expressed genes between two groups with fold change > 2. Furthermore, reactome pathway analysis was performed to elucidate the biological functions of the different pathways based on the Reactome pathway Database (http://www.reactome.org/).

### Statistical analysis

Figures were produced using GraphPad Prism software. Animals were randomly assigned to each group. Based on extensive experience with the mouse models of obesity and diabetes (for example, assay sensitivity, the different animal strains used, mortality rate) and given the planned analytical framework, we estimated the number of mice per group that would be required to detect effects of interest at the *p* < 0.05 level of significance. The numbers of technical replicates or biological replicates (independent experiments for cell culture, or individual mouse for in vivo analyses) in each group are stated in the figure legends. All values are mean ± s.e.m. in bar graphs. For box plots, centre lines represent the median; limits represent the quartiles; whiskers represent the minimum and maximum values. An unpaired Student’s *t*-test with no assumption of equal variance was used for comparisons between two groups. For comparisons of more than two groups, ANOVA (by general linear model) was used. When the overall *F* test was significant (*p* < 0.05), post hoc comparisons using Tukey’s method of adjustment were used to assess the presence of any significant pairwise differences. Analyses were performed using GraphPad Prism 5 software (La Jolla, CA). A two-sided *p* value < 0.05 was considered statistically significant.

## Supplementary information

Supplementary Materials

Supplementary Figure 1

Supplementary Figure 2

Supplementary Figure 3

Supplementary Figure 4
