# Supplementary material for: Kdm6a suppresses the alternative activation of macrophages and impairs energy expenditure in obesity
Source: Cell Death Differ. 2020 Dec 10;28(5):1688–704. doi: 10.1038/s41418-020-00694-8 (PMC8167088; doi:10.1038/s41418-020-00694-8)
Supplement: Supplementary file 1 — Supplementary Materials [file 41418_2020_694_MOESM1_ESM.doc]

**Supplementary Materials**

**Supplementary Figure Legend**

**Supplementary Figure 1**

A. PCR analysis of the *Kdm6a* ablation in the enriched CD_3_^+^ T cells, B220^+^ B cells and CD11_b_^+^ pMΦ from indicated mice.

B. Immunoblotting assays to examine the Kdm6a proteins in the BMDMs and different tissues.

C. H&E staining and IHC images of BAT in the *Kdm6a^F/Y^* and *Kdm6a^F/Y^;Lyz2*-*Cre* mice, bar=100 μm.

D. Relative mRNA abundance of *Kdm6a* in the LPS or Il4-stimulated BMDMs.

E. Immunoblotting assays to indicate Kdm6a proteins in the LPS or Il4-stimulated BMDMs.

F. Immunoblotting assays to verify the transfection of vector, full-length Kdm6a and Kdm6a truncation lacking the JmjC domain.

**Supplementary Figure 2**

A. Genome browser view of normalized ChIP-Seq signals of H3K27me3 at the *Arg1, Ym1, Retnla and Pdcd1lg2* loci in vector, OE-Kdm6a and OE-del JmjC-transfected BMDMs.

B. The scheme for cellular differentiation of 3T3-L1 cells into white adipocyte with the treatment of different type of conditional medium from BMDMs, affiliated to Fig. 7E, 7F, 8B, 8C and Supplementary Fig. 3A, 3B, 3C, 4A and 4B.

C. The scheme for detection on the cellular sensitivity of already differentiated 3T3-L1 white adipocyte with the treatment of different type of conditional medium from BMDMs, affiliated to Fig. 7G, 8D and Supplementary Fig. 4C.

D. The scheme for cellular differentiation of C3H10-T1/2 cells into brown adipocyte with the treatment of different type of conditional medium from BMDMs, affiliated to Fig. 7H, 7I, 7J, 8E, 8F, 8G and Supplementary Fig. 3D, 3E, 3F, 4D, 4E and 4F.

E. The scheme for detection on the thermogenesis of already differentiated C3H10-T1/2 brown adipocyte with the treatment of different type of conditional medium from BMDMs, affiliated to Fig. 7K, 7L, 8H and 8I.

**Supplementary Figure 3**

A. Representative oil red staining images of white adipocytes differentiation, bar = 200 μm.

B. Relative mRNA abundance of shown mRNAs in the 3T3-L1 cells. During the differentiation, cells were incubated with Il10 in the culture medium.

C. Immunoblotting assays to detect Acc, C/ebpβ and Pparγ proteins in 3T3-L1 cells.

D. Representative oil red staining images of brown adipocytes differentiation, bar = 200 μm.

E. Relative mRNA abundance of shown mRNAs in the C3H10-T1/2 cells.

F. Immunoblotting assays to examine the Ucp1 and Prdm16 proteins in the C3H10-T1/2 cells.

**Supplementary Figure 4**

A. Representative oil red staining images of white adipocytes differentiation, bar = 200 μm.

B. Immunoblotting assays to detect Acc, C/ebpβ, Pparγ, and Fabp4 proteins in 3T3-L1 cells after the treatment of indicated BMDMs conditional medium.

C. Immunoblotting assays to examine the p-Insulin Receptor and p-Akt levels in 3T3-L1 cells in response to insulin after the treatment of indicated BMDMs conditional medium.

D. Representative oil red staining images of brown adipocytes differentiation, bar = 200 μm.

E. Immunoblotting assays to examine the Ucp1 and Prdm16 proteins in the C3H10-T1/2 cells. During the differentiation, cells were incubated with conditional medium from indicated BMDMs.

F. Relative abundance of shown mRNAs in the C3H10-T1/2 cells. During the differentiation, cells were incubated with conditional medium from in indicated BMDMs medium.

Supplementary Table1

| Ab for immunoblotting | Source | Catalog Number |
| --- | --- | --- |
| Kdm6a | Cell Signaling Technology | 33510 |
| βActin | proteintech | 60008-1-lg |
| β-Tublin | Cell Signaling Technology | 2148S |
| Ucp1 | abcam | ab10983 |
| H3 | Cell Signaling Technology | 4620S |
| H3K27me3  Pparγ  Cebpβ  Prdm16  Pgc-1α  phosphor-Akt (Ser473)  Akt  Insulin receptor  Phospho-Insulin receptor (Thr1160)  Fabp4    Acetyl-CoA Carboxylase | Cell Signaling Technology  Santa Cruz  Santa Cruz  Abcam  Calbiochem  Cell Signaling Technology  Cell Signaling Technology  Santa Cruz  PhosphoSolutions  Cell Signaling Technology  Cell Signaling Technology | 9733  sc-7273  sc-7962  ab106410  ST1202  #4060  #4691  sc-711  p168-1160  2120S  3662s |

| Ab for FACS | Source | Catalog Number |
| --- | --- | --- |
| PE-anti-F4/80 | MULTI Sciences | AM048004-250 |
| FITC-anti-CD11b | Bio Legend | 101206 |
| FITC-anti-CD11c | BD | 561045 |
| APC-anti-CD11c | MULTI Sciences | AM011C05-250 |
| APC-anti-CD206 | Bio Legend | 141707 |

Supplementary Table2

| Gene | Forward primer (5'-3') | Reverse primer (5'-3') |
| --- | --- | --- |
| 18sRNA | AGGGGAGAGCGGGTAAGAG | GGACAGGACTAGGCGGAACAACA |
| Cox5a | GGAAGACCCTAATCTAGTCCCG | GTTGGGGCATCGCTGACTC |
| Kdm6a | CGGGCGGACAAAAGAAGAAC | CATAGACTTGCATCAGATCCTCC |
| Cox7a | GCTCTGGTCCGGTCTTTTAGC | GTACTGGGAGGTCATTGTCGG |
| Cox8b | TGTGGGGATCTCAGCCATAGT | AGTGGGCTAAGACCCATCCTG |
| Pparg | GGAAGACCACTCGCATTCCTT | GTAATCAGCAACCATTGGGTCA |
| Prdm16 | TGCTGACGGATACAGAGGTGT | CCACGCAGAACTTCTCGCTAC |
| Ucp1 | CAAAAACAGAAGGATTGCCGAAA | TCTTGGACTGAGTCGTAGAGG |
| Pgc1a | TATGGAGTGACATAGAGTGTGCT | CCACTTCAATCCACCCAGAAAG |
| Ccl2 | TTAAAAACCTGGATCGGAACCAA | GCATTAGCTTCAGATTTACGGGT |
| Nos2 | ACATCGACCCGTCCACAGTAT | CAGAGGGGTAGGCTTGTCTC |
| Arg1 | CTCCAAGCCAAAGTCCTTAGAG | GGAGCTGTCATTAGGGACATCA |
| Retnla | CCAATCCAGCTAACTATCCCTCC | ACCCAGTAGCAGTCATCCCA |
| Il6 | CCACGGCCTTCCCTACTTC | TTGGGAGTGGTATCCTCTGTGA |
| Ym1 | ACTGATAGCAGTTTGCCCAAG | TCTACGTTCCCCAAGTCGTTAG |
| Pdcd1lg2  Fabp4  C/ebpβ | CTGCCGATACTGAACCTGAGC  AAGGTGAAGAGCATCATAACCCT  TCGGGACTTGATGCAATCC | GCGGTCAAAATCGCACTCC  TCACGCCTTTCATAACACATTCC  AAACATCAACAACCCCGC |
| Kdm6a genotyping | AACAAAAACCCAGGCTTTATTCAC | AGTTTCAGGATACCTTTACTATAAG |
| Cre genotyping  Ire1α  Il10  Ire1α-a  Ire1α-b  Ire1α-c  Ire1α-d  Ire1α-e  Ire1α-f  Ire1α-g  Ire1α-h | ATTTGCCTGCATTACCGGTCG  ACACTGCCTGAGACCTTGTTG  CTTACTGACTGGCATGAGGATCA  ATGGCTGAGTGGTTTCCACC  AGGTCTCGTCAGCTCACCTT  AGATGCTGGCTTTGGGTCAG  CTAAGAGGGCTTCTGGGTGTG  GGTGACCCTGTTTAGACCCTG  ACCACAGACTTTGTAGGCCA  GTACCAAAAATCGCCATTCCCT  CTCCGGGAGTCAGGGTATGT | CAGCATTGCTGTCACTTGGTC  GGAGCCCGTCCTCTTGCTA  GCAGCTCTAGGAGCATGTGG  TCACAGCAGCAACCAACAGA  CGCTACAGAGCTACCCTGAC  CAGTCTCGAGATGGCCTAGTG  CCAGGGTCTAAACAGGGTCAC  ACCTCTCAGCCACTGACAAA  TCAAGGGAATGGCGATTTTTGG  ATCTCCCCCACATACCCTGA  CTCCCCACGGCTACACAATA |
